# Comment on: Need for Primary Prevention for Skin Cancers in Iran

**Published:** 2016-08-21

**Authors:** Vinayak K Nahar, Amer Hosain, Manoj Sharma, Stephanie K Jacks, Robert T Brodel

**Affiliations:** ^a^ Department of Dermatology, School of Medicine, University of Mississippi Medical Center, Jackson, MS, USA; ^b^ Department of Health, Physical Education, and Exercise Science, School of Allied Health Sciences, Lincoln Memorial University, Harrogate, TN, USA; ^c^ Biomedical Professionals, Lincoln Memorial University, Harrogate, TN, USA; ^d^ Behavioral & Environmental Health, School of Public Health, Jackson State University, MS, USA

## Dear Editor-in-Chief

Incidence rates are increasing in many countries, including Iran where skin cancers are the most commonly diagnosed cases^1^. In the southeast of Iran, nearly 15% of all diagnosed cancers were related to the skin^2^. In Yazd, east western Iran, the incidence rates showed a mean of 28.6% over a 15-yr period from 1987 to 2001^3^, during which, 1,124 patients were diagnosed with skin cancers, with 2.7% malignant melanoma, as the most life-threatening type of skin cancer.

The majority of skin cancers can be attributed to exposure to ultraviolet radiation (UVR) emitted from the sun.^4^ However, the risk of skin cancers could be greatly reduced by improved sun protection strategies (i.e., limiting direct sun exposure, using protective clothing and sunscreen)^5^. Each country can serve as a testing ground for efforts to prevent skin cancer. The strategies require that correct knowledge, positive attitudes, effective skills, and preemptive behaviors be incorporated in everyday life.

An Iranian-based study targeting 400 adults reported that a majority was well-informed about sun protection practices^6^. However, only 15% of the participants were concerned about being affected by skin cancers, and about 60% stated that use of sunscreen was difficult. With regard to sun safety, only 41% used protective clothing, and 32% reported using sunscreen as a protection against sun exposure. In another study involving 201 female college students in Iran University of Medical Sciences, just one percent of participants always wore a hat with a brim, 3.5% wore gloves, and 15.9% wore sunglasses when out in the sun^7^. A study with rural farmers (n = 238) in the northwestern part of Iran showed that the level of skin cancer preventative behavior was dismally low, indicating that less than a quarter (22%) of the farmers practice sun protection in their daily work^8^. The vast majority of the farmers (88.2%) did not perceive that their job made them more susceptible to skin cancer. One disturbing finding was that a little over two-thirds (67.6%) of the farmers agreed/totally agreed with the statement “using sunscreen had no effect on skin cancer prevention,” which pointed to a need for greater education about the harmful effects of UVR in this population.

Primary prevention should be the key element of cancer prevention and control efforts. Educational and policy interventions are strong weapons for primary prevention of skin cancers and must be advocated by the health care professionals in Iran. Newer theories such as the multi-theory model of health behavior change, must be reified to develop effective educational interventions that motivate individuals to adopt sun protection behaviors in vulnerable target populations^9^. This approach focuses on the attitudes, skills, and emotions of the individual, as well as environmental attributes that pertain to physical and social milieu. This model has shown high predictive potential with related behaviors^9^.

Health care providers may need to collaborate with media for large-scale dissemination of sun protection information. Moreover, health care professionals should actively promote effective sun protection, especially among those who are at future risk of skin cancers. While designing interventions, professionals should take into account which strategies have proven successful at increasing skin cancer knowledge and preventive mechanisms. There is compelling evidence that interventions focusing on the appearance-damaging effects of UVR have been effective in enhancing sun protection behaviors^10^. Individuals may not fear skin cancer, but as they mature, they want to avoid appearing “old.” Considering the variability in sun safety practices among different segments of the Iranian population, tailored interventions are recommended to target particular sun protection needs of these different groups.

## Conflict of Interest

None of the researchers will be benefitting financially from the study or the publication. Vinayak K. Nahar M.D., M.S., Ph.D.,Amer Hosain, M.S., Manoj Sharma Ph.D., Stephanie K. Jacks M.D.have no conflict of interests to report. Robert T. Brodell M.D. discloses the following potential conflicts of interest: Honoraria have been received from presentations for Allergan, Galderma, and PharmaDerm, a division of Nycomed US Inc. Consultant fees have been received from Galderma Laboratories, L.P. Clinical trials have been performed for Genentech and Janssen Biotech Inc. The material in this article is not believed to be relevant to any of these reported conflicts

## References

[1] Pakzad R, Soltani S, Salehiniya H (2015). Epidemiology and trend in skin cancer mortality in Iran. J Res Med Sci.

[2] Keyghobadi N, Rafiemanesh H, Mohammadian-Hafshejani A, Enayatrad M, Salehiniya H (2015). Epidemiology and trend of cancers in the province of Kerman: southeast of Iran. Asian Pac J Cancer Prev.

[3] Noorbala MT, Kafaie P (2007). Analysis of 15 years of skin cancer in central Iran (Yazd). Dermatol Online J.

[4] Skin Cancer Foundation. Skin Cancer Facts and Statistics. ACF website; 2016 [updated 8 June 2016, cited 15 July 2016] available from: http://www.skincancer.org/skin-cancer-information/skin-cancer-facts.

[5] Nahar VK, Ford MA, Jacks SK, Thielen SP, Johnson AK, Brodell RT (2015). Sun-related behaviors among individuals previously diagnosed with non-melanoma skin cancer. Indian J Dermatol Venereol Leprol.

[6] Mousavi F, Vaseie M, Vaseie L, Khajeh-Kazemi R (2011). Knowledge, attitude, and practice of adults to the protective actions against sun in northwest Tehran, Iran. Arch Iran Med.

[7] Dehbari SR, Dehdari T, Dehdari L, Mahmoudi M (2014). Predictors of Sun-Protective Practices among Iranian Female College Students: Application of Protection Motivation Theory. Asian Pac J Cancer Prev.

[8] Babazadeh T, Nadrian H, Banayejeddi M, Rezapour B. Determinants of skin cancer preventive behaviors among rural farmers in Iran: an application of protection motivation theory. J Cancer Educ. [Epub ahead of print]. 10.1007/s13187-016-1004-7 26922176

[9] Nahar VK, Sharma M, Catalano HP, Ickes MJ, Johnson P, Ford MA (2016). Testing multi-theory model (MTM) in predicting initiation and sustenance of physical activity behavior among college students. Health Promot Perspect.

[10] Williams AL, Grogan S, Clark-Carter D, Buckley E (2013). Appearance-based interventions to reduce ultraviolet exposure and/or increase sun protection intentions and behaviors: a systematic review and meta-analyses. Br J Health Psychol.

